# Distinct Second Primary Tumor Phenotypes in Oral Squamous Cell Carcinoma According to Exposure Status and Immune Background

**DOI:** 10.3390/jcm15041563

**Published:** 2026-02-16

**Authors:** Marko Tarle, Marina Raguž, Koraljka Hat, Igor Čvrljević, Ivan Salarić, Ivica Lukšić

**Affiliations:** 1 Department of Maxillofacial and Oral Surgery, Dubrava University Hospital, 10000 Zagreb, Croatia; tarlemarko1@gmail.com (M.T.); koraljkahat@gmail.com (K.H.); igor.cvrljevic@gmail.com (I.Č.); salaric@sfzg.unizg.hr (I.S.); 2 School of Dental Medicine, University of Zagreb, 10000 Zagreb, Croatia; 3 Department of Neurosurgery, Dubrava University Hospital, 10000 Zagreb, Croatia; marinaraguz@gmail.com; 4 School of Medicine, Catholic University of Croatia, 10000 Zagreb, Croatia; 5 School of Medicine, University of Zagreb, 10000 Zagreb, Croatia

**Keywords:** oral squamous cell carcinoma, second primary tumors, non-smoking non-drinking (NSND), oral lichen planus, field cancerization

## Abstract

**Background**: Second primary tumors (SPTs) are a major survivorship challenge in oral squamous cell carcinoma (OSCC), yet their biological phenotypes may differ according to exposure status and immune background. **Methods**: In this retrospective cohort (2011–2020), 242 surgically treated primary OSCC patients were classified as non-smoking, non-drinking (NSND; never smokers/never drinkers) or smoking and/or drinking (SD; any history of smoking and/or alcohol consumption). SPTs were categorized as extra-oral SPTs (eoSPTs) or multifocal oral SCC (mOSCC), with mOSCC (≥3) denoting ≥3 oral primaries. Immune background was assessed by documenting immune-modulating conditions (including oral lichen planus as an immune-mediated mucosal disorder). Multivariable logistic regression was used to evaluate predictors of eoSPTs and mOSCC. **Results**: SPT occurred in 82/242 (33.9%), comprising 54 eoSPT (22.3%) and 28 mOSCC (11.6%). Overall SPT prevalence was similar in NSND and SD patients (29.8% vs. 36.1%), but phenotype composition differed significantly (chi-square *p* = 0.004): eoSPTs were more common in SD (27.8% vs. 11.9%), whereas mOSCC was more common in NSND (17.9% vs. 8.2%); mOSCC (≥3) occurred in 10.7% of NSND versus 1.3% of SD patients. Immune-modulating conditions were associated with mOSCC but not eoSPTs. Within the immune-modulating spectrum, OLP showed strong phenotype specificity (0/20 eoSPTs; mOSCC in 7/20 [35.0%), particularly among NSND patients (38.9% with OLP vs. 12.1% without). In adjusted models, NSND status was associated with lower odds of eoSPT (OR 0.37, 95% CI 0.15–0.96), while OLP independently predicted mOSCC (OR 3.47, 95% CI 1.04–11.52). **Conclusions**: SPTs in OSCC comprise distinct phenotypes: SD patients predominantly develop eoSPTs consistent with carcinogen-associated aerodigestive field effects, whereas NSND patients exhibit an immune-associated, oral-restricted pattern with frequent mOSCC, supporting phenotype-tailored surveillance.

## 1. Introduction

Oral squamous cell carcinoma (OSCC) is the most common malignancy of the oral cavity and a major contributor to the global head and neck cancer burden, accounting for over 90% of oral cancers [1,2]. According to GLOBOCAN 2022 estimates, cancers of the lip and oral cavity cause approximately 390,000 new cases and 188,000 deaths worldwide each year, underscoring the persistent global impact of OSCC [3]. Although overall incidence declined in several Western populations during the late twentieth century, recent population-based analyses from the Surveillance, Epidemiology, and End Results (SEER) Program and the National Program of Cancer Registries (NPCR) show a reversal of this trend, with increasing incidence driven mainly by cancers of the oral tongue and a disproportionate rise among women, despite declining exposure to traditional risk factors such as tobacco and alcohol [4,5,6]. As survival improves, late oncologic events have become a central determinant of long-term outcomes. Among these, second primary tumors (SPTs) represent a particularly important survivorship challenge, with population-based studies reporting substantially elevated risks compared with the general population [7]. In a large Danish population-based cohort, 23% of OSCC patients developed a second primary cancer, corresponding to a fourfold increased risk relative to the background population (standardized incidence ratio 4.13), with the highest excess observed in the head and neck region (SIR > 40) and a median latency of 4.4 years from index OSCC to first SPT [8]. These findings highlight the sustained and clinically meaningful risk of SPT following OSCC and underscore the importance of long-term, risk-adapted surveillance strategies.

SPTs are a well-recognized feature of head and neck squamous cell carcinoma survivorship and are most commonly interpreted through the paradigm of field cancerization, in which chronic exposure to carcinogens—particularly tobacco and alcohol—induces diffuse molecular injury across the upper aerodigestive tract mucosa. Consistent with this model, SPTs arise predominantly at aerodigestive sites, most frequently within the head and neck region, followed by the lung and esophagus [9]. A large systematic review of more than 456,000 patients reported a mean overall SPT incidence of 13.2%, comprising 5.3% synchronous and 9.4% metachronous tumors, with risk accumulating over time and persisting well beyond completion of curative-intent treatment [10]. These observations reinforce the concept that SPTs represent a central and enduring survivorship risk rather than incidental findings.

At the same time, there is growing recognition that a significant proportion of OSCC occurs in patients without traditional risk factors, commonly referred to as non-smoking, non-drinking (NSND) individuals [10,11]. NSND OSCC is consistently identified as a clinically distinct subset, characterized by a higher proportion of female patients and a predominance of tongue tumors, while its carcinogenic mechanisms remain incompletely defined [11]. In contrast to tobacco- and alcohol-associated disease, which provides a coherent explanatory framework for aerodigestive field cancerization, NSND OSCC likely involves etiologically heterogeneous pathways [10,11]. Importantly, NSND patients may also have a distinct survivorship risk profile: in a recent cohort of surgically treated OSCC, van der Aa et al. reported a significantly higher risk of second primary tumors in NSND compared with SD patients in multivariable analysis (adjusted HR 3.92, 95% CI 1.23–12.48) [12]. However, across studies, estimates vary partly due to definitional heterogeneity, as many reports combine biologically and anatomically distinct events under a single “SPT” endpoint, thereby obscuring phenotype-specific patterns and weakening etiologic inference [11,12].

An important source of heterogeneity is the aggregation of extra-oral second primary tumors (eoSPTs) and multifocal oral squamous cell carcinoma (mOSCC) into a single composite outcome. Population-based data show that second primary tumors following OSCC have distinct anatomic and temporal patterns: extra-oral primaries cluster mainly in the head and neck region and lung, while a substantial proportion of second events occur within the oral cavity itself [7,8]. Biologically, eoSPTs are most plausibly explained by carcinogen-driven aerodigestive field cancerization, whereas mOSCC represents a distinct oral-restricted phenotype related to local mucosal susceptibility [10,13]. Failure to distinguish these entities risks conflating biologically divergent carcinogenic processes and may obscure exposure-specific and clinically meaningful associations [7,13].

In NSND patients, immune-mediated and chronic inflammatory mucosal conditions may preferentially promote oral-restricted multicentric carcinogenesis. Immune-modulating comorbidities, particularly oral lichen planus (OLP), are characterized by persistent immune activation and epithelial stress, and have been consistently associated with a higher burden of multiple or sequential oral primary events rather than extra-oral malignancies [13,14]. Recent surgical series of OSCC arising in the context of OLP report markedly increased rates of multiple primary presentations and repeated oncologic events, supporting the concept of an immune-associated oral field effect predominantly confined to the oral cavity.

Accordingly, the present study explicitly distinguishes eoSPTs from mOSCC to evaluate whether SPT phenotype—extra-oral versus oral-restricted multifocal disease—differs by exposure status, immune background, and age in a surgically treated OSCC cohort.

## 2. Materials and Methods

### 2.1. Study Design and Patient Cohort

This retrospective observational cohort study included consecutively treated patients with histopathologically confirmed primary oral squamous cell carcinoma (OSCC) managed at the Department of Maxillofacial Surgery, Dubrava University Hospital, Zagreb, Croatia (study period: 2011–2020). Adult patients undergoing primary surgical treatment with curative intent were eligible. Patients were excluded if documentation was insufficient to determine exposure status (smoking/alcohol), second primary tumor (SPT) outcomes, or key clinical variables required for the present analyses. Demographic and tumor-related data were extracted from institutional electronic medical records, operative documentation, and pathology reports. Baseline variables included age at diagnosis and gender. Primary OSCC subsite within the oral cavity was categorized as tongue, mandibular gingiva, retromolar trigone, maxillary gingiva, floor of mouth, buccal mucosa, vestibule, and hard palate. Pathological staging followed AJCC/UICC TNM criteria (8th edition) and was recorded as stage I–IV (IVa/IVb). Additional routinely collected histopathological variables (e.g., depth of invasion, margin status, and adverse risk features) were recorded where available but were not the principal focus of the current SPT/mOSCC analyses. Data on pre-existing oral potentially malignant disorders (e.g., oral leukoplakia or proliferative verrucous leukoplakia) were not systematically recorded in the electronic medical record over the study period and were therefore not included in the baseline tables or regression analyses.

### 2.2. Exposure Definition, Outcomes, and Immune Background

Exposure status was defined at the time of OSCC diagnosis based on the lifetime history documented in the standardized admission/preoperative medical record. Smoking and alcohol consumption were recorded as binary variables (yes/no), reflecting any history of regular tobacco use and/or alcohol consumption (current or former). Patients were classified as non-smoking, non-drinking (NSND) only if the record explicitly indicated never smoking and never drinking; all remaining patients (including former smokers and/or drinkers) were classified as smoking and/or drinking (SD).

The primary outcomes were Second Primary Tumors (SPTs) and their phenotypes. SPTs were defined as additional primary tumors distinct from the index OSCC, diagnosed synchronously or metachronously, based on clinical and histopathological documentation. Because multiple independent oral primaries represent a distinct clinical entity within OSCC survivorship, SPTs were explicitly categorized into: extra-oral SPT (eoSPT) and multifocal OSCC within the oral cavity (mOSCC). eoSPTs were defined as SPTs arising outside the oral cavity; for phenotype-based analyses, eoSPTs were evaluated using a prespecified mutually exclusive hierarchical classification to ensure non-overlapping outcome categories, whereby patients with mOSCC were not included in the eoSPT category. mOSCC was defined as the occurrence of multiple distinct primary OSCCs within the oral cavity and was recorded categorically as: no mOSCC, mOSCC (two primary oral OSCCs), and mOSCC (≥3) (three or more primary oral OSCCs).

Additional OSCCs were adjudicated as distinct second primary tumors (mOSCC) rather than local recurrence or persistent disease using a predefined, chart-based adjudication approach based on multidisciplinary clinical documentation. An oral cavity event was classified as an SPT if all of the following criteria were fulfilled: (a) occurrence in a different oral subsite than the index tumor and any prior oral cavity lesion, based on detailed clinical mapping and operative reports; (b) independent histopathological confirmation of invasive squamous cell carcinoma for each event; and (c) multidisciplinary team documentation (clinical examination, operative reports, and/or imaging) supporting a new mucosal primary lesion rather than regrowth from the index tumor bed. Events detected within 6 months of index OSCC diagnosis were classified as synchronous, whereas events detected >6 months after index diagnosis were classified as metachronous. Lesions arising in the same oral subsite with clinical or imaging features suggestive of tumor persistence or regrowth were classified as local recurrence or persistent disease and were not counted as SPTs. Molecular clonality analyses were not available.

SPT anatomic site was coded using a predefined scheme, including lung; head and neck; gastrointestinal sites (e.g., esophagus, stomach, pancreas, liver, colorectal); prostate; breast; kidney; cervix; hematologic malignancies (e.g., leukemia, multiple myeloma, myeloproliferative neoplasia); and other specified sites. For head and neck eoSPT, subsites were recorded when available (e.g., larynx, oropharynx, hypopharynx, nasopharynx, thyroid). SPT site coding was verified through record review, and unspecified subsites were resolved when sufficient documentation was available. SPT ascertainment was performed within a structured institutional follow-up framework. At our center, patients with OSCC are systematically followed from the time of diagnosis throughout their lifetime, with more frequent follow-up visits during the first years after primary treatment and progressively longer intervals during long-term follow-up. eoSPTs were identified through structured review of the institutional electronic medical record, including cross-departmental documentation from other specialist units within the same hospital, such as otorhinolaryngology, pulmonology/thoracic surgery, gastroenterology, oncology, and radiology. Each eoSPT diagnosis was verified using available histopathological reports and/or specialist correspondence. Linkage with a national cancer registry was not performed. Post-treatment surveillance followed institutional practice, with more intensive follow-up during the first 1–2 years after treatment and progressively longer intervals thereafter, including focused head and neck examination at each visit. Imaging and additional investigations (including chest imaging and aerodigestive evaluation) were performed based on clinical indications and multidisciplinary recommendations rather than a uniform screening protocol, and suspected lesions were investigated with targeted imaging and/or biopsy.

Immune background was assessed through documentation of immune-modulating conditions, recorded as a binary variable (present/absent) and complemented by diagnosis-level coding. Immune-modulating conditions were abstracted from the baseline medical history at the time of index OSCC diagnosis, based on standardized admission documentation and specialist correspondence. Diagnoses were considered present only if documented prior to or at the index OSCC diagnosis; conditions first diagnosed after OSCC treatment were not considered baseline risk factors. Immune-modulating conditions were defined a priori to include autoimmune and chronic inflammatory diseases, immune-mediated endocrine disorders, hematologic malignancies affecting immune function, chronic viral infections, and clinical states associated with immune dysregulation or immunosuppression (e.g., organ transplantation). Where available, immune diagnoses were corroborated by specialist documentation, diagnostic coding, and/or medication history. Patients could contribute more than one immune-modulating diagnosis, and a separate binary variable captured the presence of two or more immune-modulating conditions. Oral lichen planus (OLP) was identified based on clinical documentation by treating specialists, with histopathological confirmation when available, and was analyzed separately from other immune-modulating conditions based on a prespecified biological rationale. OLP was not assigned based on administrative coding alone. For each case, OLP status was confirmed by structured review of specialist documentation (oral medicine/dermatology and/or head and neck team notes) available at or before the index OSCC diagnosis, and by histopathology reports when available. Cases documented as oral leukoplakia or as non-specific oral lichenoid lesions/reactions without a definitive OLP diagnosis were not coded as OLP.

### 2.3. Statistical Analysis

Age was analyzed both as a continuous variable and categorically using prespecified strata (≤50 vs. >50 years). Categorical variables are reported as counts and percentages; continuous variables are summarized as medians with interquartile ranges (IQR). Between-group comparisons used the chi-square test or Fisher’s exact test for categorical variables and the Mann–Whitney U test for continuous variables, as appropriate. Differences in SPT phenotype distribution (No SPT vs. eoSPT vs. mOSCC) were assessed using chi-square testing. No formal adjustment for multiple comparisons was applied; therefore, results from repeated chi-square testing and subgroup analyses should be interpreted as exploratory. Univariable and multivariable logistic regression models were fitted to identify predictors of eoSPT and mOSCC, reporting odds ratios (OR) with 95% confidence intervals (CI). Multivariable models specified a priori and included NSND status, age (per 10-year increase), gender, and immune-modulating comorbidity; the mOSCC model additionally incorporated OLP based on biological plausibility. Sensitivity analyses additionally adjusted for follow-up duration to account for differences in time at risk. All tests were two-sided, and *p*-values < 0.05 were considered statistically significant. Statistical analyses were performed using MedCalc, version 12.5.0 (MedCalc Software, Ostend, Belgium).

### 2.4. Ethical Approval

This study was approved by the Ethics Committee of Dubrava University Hospital (2024/1022-5, 22 October 2024). The requirement for written informed consent was waived due to the retrospective design and use of routinely collected clinical data. All data were anonymized prior to analysis. The study was conducted in accordance with the Declaration of Helsinki and applicable data protection regulations.

## 3. Results

A total of 242 surgically treated primary OSCC patients were analyzed, including 84 (34.7%) classified as NSND and 158 (65.3%) as SD. The median age at diagnosis was 50.5 years (IQR 46.0–64.0), and 70 patients (28.9%) were female. Median follow-up was 60 months (IQR 16–102), with follow-up extending up to 217 months, reflecting substantial long-term observation. Follow-up duration did not differ significantly between NSND and SD patients but was longer among patients with oral lichen planus (Supplementary Table S1). Stage distribution (I–IV) was comparable between NSND and SD patients (*p* = 0.676) and therefore unlikely to fully explain observed phenotype differences. Immune-modulating comorbidities were documented in 85 patients (35.1%) and were markedly enriched in NSND patients (67.9%) compared with SD patients (17.7%; *p* < 0.001), driven primarily by oral lichen planus (OLP) and endocrine immune conditions (Table 1).

**Figure 1:**
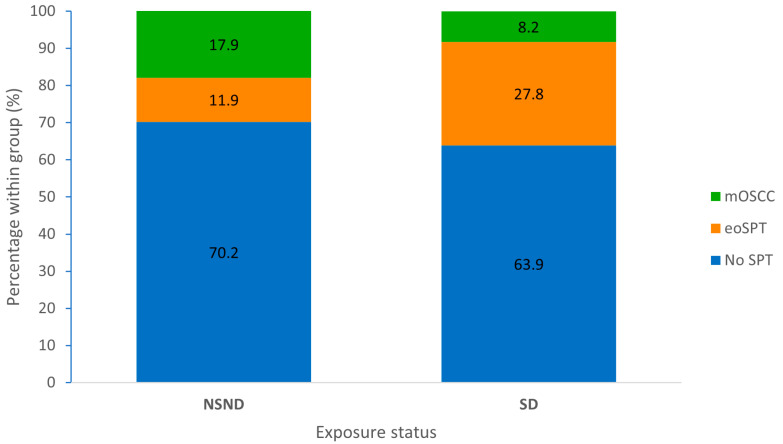
Second primary tumor patterns by exposure status. Stacked bar chart illustrating the distribution of SPT phenotypes according to exposure status. Bars represent the percentage of patients within each exposure group classified as having no SPT, eoSPT or mOSCC. NSND patients show a higher relative proportion of mOSCC, whereas SD patients predominantly develop eoSPTs.

Overall, SPTs were observed in 82 patients, corresponding to 33.9% of the cohort. After explicit separation of mOSCC from eoSPTs, 54 patients (22.3%) developed eoSPTs and 28 (11.6%) developed mOSCC (Table 2). Phenotypic overlap was uncommon: three patients (1.2%) developed both mOSCC and an eoSPT (Supplementary Table S2). While the overall prevalence of any SPT did not differ significantly between NSND and SD patients (29.8% vs. 36.1%; *p* = 0.392), the composition of SPTs differed substantially between exposure groups (chi-square *p* = 0.004; Figure 1). eoSPTs were significantly more frequent in SD patients than in NSND patients (27.8% vs. 11.9%; *p* = 0.005), whereas mOSCC was more frequent in NSND patients (17.9% vs. 8.2%; *p* = 0.034). Notably, the occurrence of mOSCC (≥3) was markedly more frequent in NSND patients (10.7% vs. 1.3%; *p* = 0.002).

**Figure 2:**
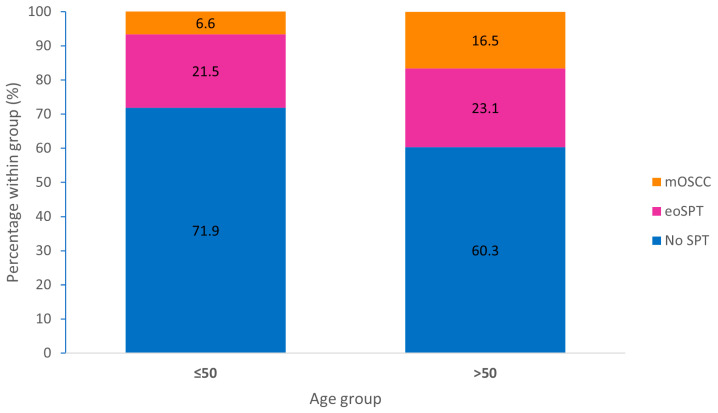
Second primary tumor patterns by age group. Stacked bar chart showing the distribution of second primary tumor (SPT) phenotypes stratified by age (≤50 vs. >50 years). Bars depict the percentage of patients within each age group with no SPT, eoSPT, or mOSCC. The age-related shift in SPT composition is driven primarily by an increased prevalence of mOSCC in patients older than 50 years.

Age stratification suggested a non-significant trend toward a higher overall prevalence of SPTs in patients older than 50 years compared with those aged ≤50 years (39.7% vs. 28.1%; *p* = 0.077). This age-related difference was driven primarily by an increased prevalence of mOSCC (16.5% vs. 6.6%; *p* = 0.026), rather than eoSPTs (23.1% vs. 21.5%; *p* = 0.877), resulting in a significant shift in SPT composition across age groups (chi-square *p* = 0.040; Figure 2). Among NSND patients, the prevalence of SPT increased from 17.1% in those aged ≤50 years to 38.8% in those older than 50 years (*p* = 0.052), whereas SPT prevalence in SD patients was comparable across age strata (32.6% vs. 40.3%; *p* = 0.324) (Table 3).

Among eoSPTs with documented anatomic site, the most frequent localizations were the head and neck region (38.9%) and the lung (20.4%), followed by the prostate, esophagus, and kidney (each 5.6%) (Table 4). Head and neck eoSPTs occurred predominantly in SD patients (18/21, 85.7%); among cases with a specified head and neck subsite, the larynx, oropharynx, and hypopharynx each accounted for approximately one third of SD cases (Table 5). In contrast, head and neck eoSPTs were uncommon in NSND patients and showed a heterogeneous subsite distribution (Table 5).

**Figure 3:**
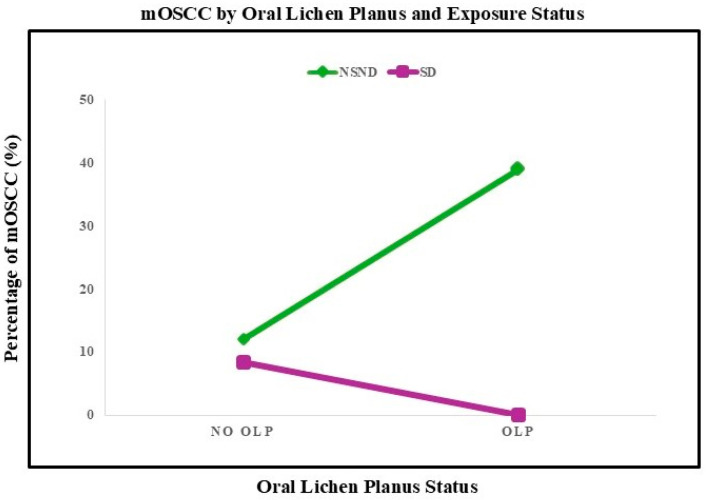
Association between oral lichen planus and mOSCC stratified by exposure status. Line plot illustrating the prevalence of multifocal oral squamous cell carcinoma (mOSCC) according to oral lichen planus (OLP) status in non-smoking, non-drinking (NSND) and smoking and/or drinking (SD) patients. Among NSND patients, the presence of OLP was associated with a marked increase in mOSCC prevalence, whereas no corresponding increase was observed in SD patients. Values represent the percentage of patients within each exposure group.

Immune-modulating comorbidity, considered as an aggregate, was not associated with the occurrence of any SPT (*p* = 0.256) or eoSPT (*p* = 0.628), but was significantly associated with mOSCC (57.1% in patients with immune-modulating disease vs. 32.2% in those without; *p* = 0.012) (Table 6). OLP demonstrated the clearest phenotype specificity: no patient with OLP developed eoSPTs (0/20), whereas mOSCC occurred in 7/20 (35%) patients with OLP (Figure 3). Among NSND patients, mOSCC was observed in 38.9% of those with OLP compared with 12.1% of those without OLP (*p* = 0.015).

In multivariable logistic regression, NSND status remained associated with lower odds of eoSPTs after adjustment for age, gender, and immune-modulating comorbidity (OR 0.37, 95% CI 0.15–0.96; *p* = 0.041), whereas increasing age was associated with higher odds of eoSPTs (OR 1.34 per 10 years, 95% CI 1.03–1.74; *p* = 0.031) and female gender was independently protective (OR 0.23, 95% CI 0.08–0.66; *p* = 0.006) (Table 7). For mOSCC, increasing age (OR 1.37 per 10 years, 95% CI 1.01–1.84; *p* = 0.042) and OLP (OR 3.47, 95% CI 1.04–11.52; *p* = 0.043) emerged as independent predictors, whereas NSND status was not independently associated after adjustment (Table 7).

## 4. Discussion

Second primary tumors (SPTs) are among the most clinically significant long-term challenges in oral squamous cell carcinoma (OSCC) survivorship, as they increase diagnostic complexity, often require additional therapeutic interventions, and may ultimately affect outcomes related to the index tumor. Population-based studies consistently show that the risk of developing SPTs after OSCC remains persistently elevated compared with the general population, with a continuous incidence observed throughout long-term follow-up, regardless of the time elapsed since primary treatment [15]. Several contemporary studies have further reported that NSND patients may have a higher overall risk of SPTs when second primaries are analyzed as a single aggregated endpoint [12]. However, such aggregate analyses do not distinguish between biologically distinct SPT phenotypes. Our findings indicate that, while the overall burden of SPTs may appear similar across exposure groups, the underlying phenotype differs substantially: NSND patients predominantly develop oral-restricted multifocal disease, whereas SD patients are more frequently affected by eoSPTs. This distinction reconciles apparent discrepancies across studies and suggests that the increased SPT risk reported in NSND populations is driven primarily by oral multifocality rather than a generalized predisposition to extra-oral malignancies. Clinically, this differentiation is highly relevant, as similar overall SPT rates may result from fundamentally different carcinogenic mechanisms and therefore require distinct, phenotype-informed surveillance strategies.

In the present cohort, 33.9% of patients developed an SPT phenotype during follow-up (median 60 months, IQR 16–102; maximum 217 months). This estimate is higher than the mean incidence reported in large systematic reviews of head and neck cancer (≈13% with minimum follow-up ≥22 months) [10], and higher than several population-based OSCC cohorts reporting approximately 20–25% second primaries over long-term follow-up [8]. Several factors may explain the higher observed incidence. First, our phenotype-based endpoint explicitly includes oral-restricted mOSCC, which is often excluded from registry-based SPT definitions or classified as local recurrence, thereby lowering reported SPT rates in those series. Second, the long right tail of follow-up in our cohort increases the cumulative probability of detecting late SPT events. Third, Dubrava University Hospital is a tertiary center with integrated follow-up pathways; systematic review of cross-departmental electronic medical records and multidisciplinary documentation may increase ascertainment of second primaries compared with studies relying on single-department follow-up. Finally, we required clinical and/or histopathological corroboration for each SPT diagnosis, but molecular clonality data were unavailable and some misclassification between recurrence and new primary cannot be entirely excluded; this limitation is discussed below.

The predominance of eoSPTs among SD patients aligns with the classical carcinogen-associated field cancerization model in head and neck oncology. Extensive epidemiological evidence shows that tobacco and alcohol exposure cause diffuse molecular injury across the upper aerodigestive tract, resulting in a persistent, long-term risk of second primary malignancies even after curative treatment of the index tumor [16,17]. Population-based studies have consistently shown that survivors of oral cavity and pharyngeal cancers have among the highest relative risks for subsequent malignancies, particularly in the head and neck region, lung, and esophagus, with standardized incidence ratios exceeding 3 and remaining elevated beyond 10 years of follow-up [16]. In surgically treated head and neck cancer cohorts, tobacco and alcohol exposure, cumulative dose intensity, and comorbidity burden have repeatedly emerged as independent predictors of eoSPT, while oral cavity sub-sites confer a comparatively lower risk of extra-oral events [18]. These findings are consistent with systematic reviews showing that toxic lifestyle habits are the most consistently associated and biologically plausible drivers of SPT development in exposure-associated disease [17]. Together, these data reinforce the interpretation that, in SD patients, SPT risk is predominantly mediated by carcinogen-driven aerodigestive field effects and persists throughout long-term survivorship [19].

In contrast, the SPT phenotype observed in NSND patients was markedly different and largely limited to the oral cavity. In a population-based cohort, NSND individuals accounted for 12.6% of head and neck cancer patients and showed a predominance of oral cavity tumors, with no second primary tumors detected during follow-up [19,20]. Similarly, Koo et al. reported that 100% of second primary tumors in NSND oral squamous cell carcinoma patients were confined to the oral cavity, whereas 20% of second primaries in patients who smoked or drank occurred at extraoral sites [21]. Notably, NSND patients also exhibited a higher burden of oral multiplicity, with 40% of those with second primaries developing more than one additional oral primary [22]. Consistent with these findings, recent reviews describe NSND OSCC as a distinct clinicobiological subset characterized by female predominance, older age at diagnosis, preferential involvement of the oral tongue and alveolar ridge subsites, and a relative paucity of lung and esophageal second primaries, despite comparable or improved 5-year survival compared with exposure-associated disease [13,20]. These data support the concept that NSND OSCC follows an oral-restricted, multifocal disease course driven by non-carcinogen pathways rather than generalized cancer susceptibility.

A key contribution of this study is the demonstration that immune background is selectively associated with mOSCC, but not with eoSPTs. Immune-modulating comorbidities were significantly linked to mOSCC, while no association was observed with eoSPTs, arguing against a model of generalized cancer susceptibility and instead supporting an immune-associated, oral-restricted field effect. In this context, chronic immune-mediated mucosal inflammation appears to promote repeated, independent malignant transformation events confined to the oral cavity.

Population-based evidence supports this interpretation, indicating that immune-mediated oral disorders confer a localized risk of OSCC without increasing extra-oral malignancy rates. In a 25-year population-based cohort, oral lichen planus (OLP) was associated with a cumulative OSCC incidence of 3.1% at 20 years, corresponding to a 4.8-fold increased risk, with all malignant events arising within the oral cavity [23]. High-quality meta-analyses using strict diagnostic criteria estimate OLP malignant transformation rates between 0.44% and 2.28%, with multifocal oral disease frequently observed among affected patients [24]. Mechanistic studies further support the biological plausibility of an immune-associated oral field effect, demonstrating early and compartment-specific immune microenvironment changes during oral carcinogenesis, including sustained T cell–rich infiltrates, cytokine signaling, epithelial stress, and macrophage-dominated immune responses at the dysplasia stage [25,26,27]. Collectively, these processes may facilitate genomic instability and immune escape, predisposing to repeated primary tumor development rather than isolated malignant transformation, a paradigm that appears particularly relevant in NSND patients.

Age emerged as an important modifier of SPT patterns. Although increasing age was associated with both eoSPTs and mOSCC, the age-related shift in SPT composition was driven primarily by an increase in oral-restricted multifocal disease. Population-based registry data indicate that the absolute risk of SPTs after OSCC peaks in patients aged 60–75 years, with a relative shift toward oral cavity SPTs in older age groups [7]. The stronger association between immune-mediated disease and mOSCC in patients older than 50 years is biologically plausible in the context of age-related immune remodeling. Aging is characterized by immunosenescence and chronic low-grade inflammation (“inflammaging”), with expansion of immunosuppressive cell populations that impair immune surveillance and promote tumor permissiveness [28]. Consistent with this framework, clinicopathological studies report that patients who develop oral SPTs are on average 5–10 years older than those without SPTs, supporting a synergistic interaction between age and immune-mediated mucosal pathology in driving oral-restricted SPT development [19].

From a clinical perspective, these findings have direct implications for post-treatment surveillance in OSCC. They argue against a uniform “SPT surveillance” approach and instead support phenotype-adapted follow-up strategies. In SD patients, surveillance should prioritize the aerodigestive tract and lungs, reflecting the dominant pattern of eoSPTs. In NSND patients, particularly those with OLP or other immune-modulating conditions, surveillance should focus intensively on the entire oral cavity, with meticulous inspection of all subsites, a low threshold for biopsy of new or evolving lesions, and close collaboration with oral medicine and dermatology specialists. This approach aligns with emerging evidence that multiple independent oral primaries are a defining feature of immune-associated OSCC phenotypes.

Several limitations should be considered. The retrospective, single-center design limits both causal inference and generalizability. Human papillomavirus (HPV) status was not systematically available, as p16 immunohistochemistry or HPV testing was not performed routinely for oral cavity OSCC during the study period; therefore, residual biological heterogeneity within the non-smoking/non-drinking subgroup cannot be excluded. In addition, OLP was ascertained retrospectively from baseline specialist documentation with histopathological confirmation when available; therefore, some degree of misclassification cannot be excluded, and any non-differential misclassification would be expected to bias associations toward the null. Baseline oral potentially malignant disorders (e.g., leukoplakia and proliferative verrucous leukoplakia) were not systematically captured in the electronic medical record over the study period, precluding reliable reporting or adjustment for these lesions. Smoking and alcohol exposure were recorded as binary variables, which precludes dose–response analyses and allows for residual confounding. Such non-differential exposure misclassification would be expected to attenuate dose–response relationships rather than exaggerate observed associations. The timing of SPTs (synchronous versus metachronous) was not systematically captured for all patients, preventing time-to-event and competing-risk analyses. Although we minimized misclassification by explicitly separating mOSCC from eoSPTs, distinguishing true second primaries from late recurrences remains challenging in routine clinical practice, and molecular clonality data were not available. Detection of eoSPTs, particularly lung and aerodigestive tract malignancies, may be influenced by differential investigation intensity (e.g., symptom-driven imaging and risk-based work-up). Although we reviewed institutional records across specialties to capture events, residual detection bias cannot be excluded. SPTs were identified through institutional medical records without linkage to a national cancer registry, which may influence the cumulative detection of SPTs compared with registry-based studies. Several immune-modulating diagnoses were rare, so interpretation should focus on phenotype-level patterns rather than individual low-frequency conditions. In this context, the observed association between immune-modulating conditions and mOSCC was largely driven by OLP, which demonstrated the clearest phenotype specificity, while other immune diagnoses were individually infrequent. In particular, the limited number of mOSCC events may have contributed to wide confidence intervals in multivariable models, and effect size estimates should therefore be interpreted with appropriate caution. Adjuvant treatment variables were not uniformly available in a structured manner in this retrospective dataset; therefore, we could not directly adjust for treatment intensity, which may influence survival, time at risk, and surveillance intensity. Despite these limitations, this study has notable strengths. The cohort represents a real-world, surgically treated OSCC population with detailed characterization of exposure status, immune background, and SPT phenotypes. The analytical strategy—explicit separation of eoSPTs from mOSCC—revealed etiologically coherent patterns that would have remained obscured if SPTs had been treated as a single endpoint. The internal consistency of findings across univariable analyses, age stratification, and multivariable models, together with concordance with contemporary literature on NSND OSCC and OLP-associated carcinogenesis, supports the robustness of the central interpretation.

## 5. Conclusions

SPT in OSCC represents an etiologically heterogeneous spectrum rather than a single clinical entity. SD patients predominantly develop eoSPTs consistent with carcinogen-associated aerodigestive field cancerization, while NSND patients exhibit an immune-associated, oral-restricted SPT phenotype characterized by multifocal oral primaries and a strong association with immune-mediated mucosal disease, particularly OLP. Recognizing this heterogeneity supports phenotype-informed surveillance and underscores the importance of integrating immune background into long-term risk stratification for OSCC survivors.

## Figures and Tables

**Table 1:** Demographics and immune background by exposure status.

Characteristic	Overall (*n* = 242)	NSND (*n* = 84)	SD (*n* = 158)	*p* (NSND vs. SD)
Age, years, median (IQR)	50.5 (46.0–64.0)	55.0 (45.0–74.0)	50.0 (46.0–61.0)	0.024
Female sex, *n* (%)	70 (28.9%)	50 (59.5%)	20 (12.7%)	<0.001
Tumor stage				0.676
I	58 (24%)	19 (22.6%)	39 (24.7)	
II	66 (27.3%)	19 (22.6%)	47 (29.7%)	
III	36 (14.9%)	11 (13.1%)	25 (15.8%)	
IVa	75 (31%)	32 (38.1%)	43 (27.2%)	
IVb	7 (2.9%)	3 (3.6%)	4 (2.5%)	
Any immune-modulating disease, *n* (%)	85 (35.1%)	57 (67.9%)	28 (17.7%)	<0.001
≥2 immune-modulating diseases, *n* (%)	23 (9.5%)	20 (23.8%)	3 (1.9%)	<0.001
Oral lichen planus (OLP), *n* (%)	20 (8.3%)	18 (21.4%)	2 (1.3%)	<0.001
Diabetes, *n* (%)	35 (14.5%)	23 (27.4%)	12 (7.6%)	<0.001
Autoimmune hypothyroidism, *n* (%)	14 (5.8%)	13 (15.5%)	1 (0.6%)	<0.001

**Table 2:** Patterns of second primary tumors according to exposure status.

Outcome	Overall (*n* = 242)	NSND (*n* = 84)	SD (*n* = 158)	*p* (NSND vs. SD)
Any SPT	82/242 (33.9%)	25/84 (29.8%)	57/158 (36.1%)	0.392
eoSPT	54/242 (22.3%)	10/84 (11.9%)	44/158 (27.8%)	0.005
mOSCC (any)	28/242 (11.6%)	15/84 (17.9%)	13/158 (8.2%)	0.034
mOSCC	17/242 (7.0%)	6/84 (7.1%)	11/158 (7.0%)	1.000
mOSCC ≥ 3	11/242 (4.5%)	9/84 (10.7%)	2/158 (1.3%)	0.002
SPT phenotype distribution				0.004

mOSCC categories denote multifocal oral squamous cell carcinoma with two primary oral tumors (mOSCC) or three or more primary oral tumors (mOSCC ≥3). The *p*-value for SPT phenotype distribution (0.004) refers to a chi-square test across three categories (No SPT, eoSPT, and mOSCC).

**Table 3:** Patterns of second primary tumors stratified by exposure status and age group.

Outcome	NSND ≤ 50 (*n* = 35)	NSND > 50 (*n* = 49)	SD ≤ 50 (*n* = 86)	SD > 50 (*n* = 72)	*p* (≤50 vs. >50) NSND	*p* (≤50 vs. >50) SD
Any SPT	6/35 (17.1%)	19/49 (38.8%)	28/86 (32.6%)	29/72 (40.3%)	0.052	0.324
eoSPT	2/35 (5.7%)	8/49 (16.3%)	24/86 (27.9%)	20/72 (27.8%)	0.182	1.000
mOSCC (any)	4/35 (11.4%)	11/49 (22.4%)	4/86 (4.7%)	9/72 (12.5%)	0.253	0.087
mOSCC	1/35 (2.9%)	5/49 (10.2%)	3/86 (3.5%)	8/72 (11.1%)	0.393	0.113
mOSCC ≥3	3/35 (8.6%)	6/49 (12.2%)	1/86 (1.2%)	1/72 (1.4%)	0.729	1.000
SPT phenotype distribution					0.096	0.192

**Table 4:** Anatomic distribution of extra-oral second primary tumors (eoSPT; mOSCC excluded).

Site	NSND (*n* = 10)	SD (*n* = 44)	Total (*n* = 54)
Head and neck	3 (30.0%)	18 (41%)	21 (38.9%)
Lung	1 (10.0%)	10 (22.7%)	11 (20.4%)
Prostate	1 (10.0%)	2 (4.5%)	3 (5.6%)
Esophagus	0 (0.0%)	3 (6.8%)	3 (5.6%)
Kidney	0 (0.0%)	3 (6.8%)	3 (5.6%)
Colorectal	1 (10.0%)	1 (2.3%)	2 (3.7%)
Multiple myeloma	0 (0.0%)	2 (4.5%)	2 (3.7%)
Liver	0 (0.0%)	2 (4.5%)	2 (3.7%)
Stomach	0 (0.0%)	1 (2.3%)	1 (1.9%)
Myeloproliferative neoplasia	1 (10.0%)	0 (0.0%)	1 (1.9%)
Breast	1 (10.0%)	0 (0.0%)	1 (1.9%)
Pancreas	0 (0.0%)	1 (2.3%)	1 (1.9%)
Leukemia	1 (10.0%)	0 (0.0%)	1 (1.9%)
Cervix	0 (0.0%)	1 (2.3%)	1 (1.9%)
Parathyroid gland	1 (10.0%)	0 (0.0%)	1 (1.9%)

**Table 5:** Head and neck subsites among extra-oral head and neck second primary tumors (eoSPT; *n* = 21).

Head and Neck Subsite	NSND (*n* = 3)	SD (*n* = 18)	Total (*n* = 21)
Larynx	0 (0.0%)	7 (38.8%)	7 (33.3%)
Oropharynx	0 (0.0%)	6 (33.3%)	6 (28.6%)
Thyroid	1 (33.3%)	0 (0.0%)	1 (4.8%)
Hypopharynx	1 (33.3%)	5 (27.9%)	6 (28.6%)
Nasopharynx	1 (33.3%)	0 (0.0%)	1 (4.8%)

**Table 6:** Immune-modulating diagnoses by SPT phenotype.

Immune-Modulating Diagnosis	No SPT (*n* = 160)	eoSPT (*n* = 54)	mOSCC (*n* = 28)
Any immune-modulating disease	52 (32.5%)	17 (31.5%)	16 (57.1%)
No immune-modulating disease	108 (67.5%)	37 (68.5%)	12 (42.9%)
≥2 immune-modulating diseases	13 (8.1%)	5 (9.3%)	5 (17.9%)
Diabetes	23 (14.4%)	7 (13.0%)	5 (17.9%)
Oral lichen planus	13 (8.1%)	0 (0.0%)	7 (25.0%)
Autoimmune hypothyroidism	9 (5.6%)	3 (5.6%)	2 (7.1%)
Psoriasis	3 (1.9%)	2 (3.7%)	1 (3.6%)
Severe allergies	3 (1.9%)	0 (0.0%)	1 (3.6%)
Immune thrombocytopenia (ITP)	2 (1.2%)	1 (1.9%)	0 (0.0%)
Vitiligo	0 (0.0%)	3 (5.6%)	0 (0.0%)
Multiple myeloma	0 (0.0%)	2 (3.7%)	0 (0.0%)
Autoimmune polyglandular syndrome 1	0 (0.0%)	0 (0.0%)	2 (7.1%)
Thrombophilia	2 (1.2%)	0 (0.0%)	0 (0.0%)
Viral hepatitis	2 (1.2%)	0 (0.0%)	0 (0.0%)
Autoimmune hyperthyroidism	2 (1.2%)	0 (0.0%)	0 (0.0%)
Rheumatoid arthritis	2 (1.2%)	0 (0.0%)	0 (0.0%)
Myeloproliferative neoplasia	1 (0.6%)	1 (1.9%)	0 (0.0%)
Dermatitis herpetiformis (celiac-related)	1 (0.6%)	0 (0.0%)	0 (0.0%)
Alopecia	2 (1.2%)	0 (0.0%)	0 (0.0%)
Ankylosing spondylitis	0 (0.0%)	0 (0.0%)	1 (3.6%)
Sarcoidosis	0 (0.0%)	1 (1.9%)	0 (0.0%)
Organ transplantation	0 (0.0%)	1 (1.9%)	0 (0.0%)
Multiple sclerosis	0 (0.0%)	1 (1.9%)	0 (0.0%)
Autoimmune hepatitis	0 (0.0%)	0 (0.0%)	1 (3.6%)
Leukemia	0 (0.0%)	1 (1.9%)	0 (0.0%)
Hyperparathyroidism	1 (0.6%)	0 (0.0%)	0 (0.0%)

**Table 7:** Multivariable logistic regression models for eoSPTs and mOSCC.

Predictor	OR (95% CI)	*p*
Outcome: eoSPT (multivariable)		
NSND vs. SD	0.37 (0.15–0.96)	0.041
Age (per 10 years)	1.34 (1.03–1.74)	0.031
Female	0.23 (0.08–0.66)	0.006
Immune-modulating disease	1.83 (0.82–4.06)	0.141
Outcome: mOSCC (multivariable)		
NSND vs. SD	1.30 (0.46–3.67)	0.625
Age (per 10 years)	1.37 (1.01–1.84)	0.042
Female	1.11 (0.38–3.20)	0.851
Oral lichen planus	3.47 (1.04–11.52)	0.043

## Data Availability

All data generated or analyzed during this study are included in this published article.

## Supplementary Materials

The following supporting information can be downloaded at: https://www.mdpi.com/article/10.3390/jcm15041563/s1, Table S1: Median follow-up duration across key strata.; Table S2: Overlap between eoSPT and mOSCC phenotypes.; Table S3: Sensitivity logistic regression models for eoSPT.; Table S4: Sensitivity logistic regression models for mOSCC.; Table S5: Exploratory mOSCC model restricted to NSND patients.

## Abbreviations

The following abbreviations are used in this manuscript:

AJCC	American Joint Committee on Cancer
eoSPT	Extra-Oral Second Primary Tumor
GLOBOCAN	Global Cancer Observatory
HNSCC	Head and Neck Squamous Cell Carcinoma
mOSCC	Multifocal Oral Squamous Cell Carcinoma
NPCR	National Program of Cancer Registries
NSND	Non-Smoking, Non-Drinking
OLP	Oral Lichen Planus
OSCC	Oral Squamous Cell Carcinoma
SD	Smoking and/or Drinking
SEER	Surveillance, Epidemiology, and End Results Program
SPT	Second Primary Tumor
TNM	Tumor–Node–Metastasis
UICC	Union for International Cancer Control

## References

[1] Al-Jamaei A.A.H., van Dijk B.A.C., Helder M.N., Forouzanfar T., Leemans C.R., de Visscher J. (2022). A population-based study of the epidemiology of oral squamous cell carcinoma in the Netherlands 1989–2018, with emphasis on young adults. Int. J. Oral Maxillofac. Surg..

[2] Tan Y., Wang Z., Xu M., Li B., Huang Z., Qin S., Nice E.C., Tang J., Huang C. (2023). Oral squamous cell carcinomas: State of the field and emerging directions. Int. J. Oral Sci..

[3] Tarle M., Luksic I. (2024). Pathogenesis and Therapy of Oral Carcinogenesis. Int. J. Mol. Sci..

[4] Patel S.C., Carpenter W.R., Tyree S., Couch M.E., Weissler M., Hackman T., Hayes D.N., Shores C., Chera B.S. (2011). Increasing incidence of oral tongue squamous cell carcinoma in young white women, age 18 to 44 years. J. Clin. Oncol..

[5] Stepan K.O., Mazul A.L., Larson J., Shah P., Jackson R.S., Pipkorn P., Kang S.Y., Puram S.V. (2023). Changing Epidemiology of Oral Cavity Cancer in the United States. Otolaryngol. Head Neck Surg..

[6] Ellington T.D., Henley S.J., Senkomago V., O’Neil M.E., Wilson R.J., Singh S., Thomas C.C., Wu M., Richardson L.C. (2020). Trends in Incidence of Cancers of the Oral Cavity and Pharynx―United States 2007–2016. MMWR Morb. Mortal Wkly Rep..

[7] Brands M.T., Campschroer G., Merkx M.A.W., Verbeek A.L.M., van Dijk B.A.C., Geurts S.M.E. (2021). Second primary tumours after squamous cell carcinoma of the oral cavity. Eur. J. Surg. Oncol..

[8] Petersen L.O., Jensen J.S., Jakobsen K.K., Gronhoj C., Wessel I., von Buchwald C. (2022). Second primary cancer following primary oral squamous cell carcinoma: A population-based, retrospective study. Acta Oncol..

[9] Bertolini F., Trudu L., Banchelli F., Schipilliti F., Napolitano M., Alberici M.P., Depenni R., D’Angelo E., Mattioli F., Rubino L. (2021). Second primary tumors in head and neck cancer patients: The importance of a “tailored” surveillance. Oral Dis..

[10] Coca-Pelaz A., Rodrigo J.P., Suarez C., Nixon I.J., Makitie A., Sanabria A., Quer M., Strojan P., Bradford C.R., Kowalski L.P. (2020). The risk of second primary tumors in head and neck cancer: A systematic review. Head Neck.

[11] Tran Q., Maddineni S., Arnaud E.H., Divi V., Megwalu U.C., Topf M.C., Sunwoo J.B. (2023). Oral cavity cancer in young, non-smoking, and non-drinking patients: A contemporary review. Crit. Rev. Oncol. Hematol..

[12] van der Aa P.J.P., Witjes M.J.H., van der Vegt B., Schuuring E., Boeve K., Sidorenkov G., de Bock G.H., de Visscher S. (2025). Non-smoking and Non-drinking Oral Cancer Patients Are at Higher Risk of Second Primary Tumours. Oral Dis..

[13] Badwelan M., Muaddi H., Ahmed A., Lee K.T., Tran S.D. (2023). Oral Squamous Cell Carcinoma and Concomitant Primary Tumors, What Do We Know? A Review of the Literature. Curr. Oncol..

[14] Yosefof E., Kurman N., Edri N., Rosenfeld E., Bachar G., Shpitzer T., Yehuda M., Mizrachi A., Najjar E. (2024). The Clinical Behavior and Recurrence Patterns of Oral Cavity Cancer in Oral Lichen Planus Patients. Laryngoscope.

[15] Mroueh R., Nevala A., Haapaniemi A., Pitkaniemi J., Salo T., Makitie A.A. (2020). Risk of second primary cancer in oral squamous cell carcinoma. Head Neck.

[16] Feller A., Matthes K.L., Bordoni A., Bouchardy C., Bulliard J.L., Herrmann C., Konzelmann I., Maspoli M., Mousavi M., Rohrmann S. (2020). The relative risk of second primary cancers in Switzerland: A population-based retrospective cohort study. BMC Cancer.

[17] Salcedo-Bellido I., Requena P., Mateos R., Ortega-Rico C., Olmedo-Requena R., Lozano-Lorca M., Arrebola J.P., Barrios-Rodriguez R. (2022). Factors associated with the development of second primary tumours in head and neck cancer patients. Eur. J. Cancer Care.

[18] Bugter O., van Iwaarden D.L.P., van Leeuwen N., Nieboer D., Dronkers E.A.C., Hardillo J.A.U., Baatenburg de Jong R.J. (2021). A cause-specific Cox model for second primary tumors in patients with head and neck cancer: A RONCDOC study. Head Neck.

[19] Karan J., Rosin M.P., Zhang L., Laronde D.M. (2024). Clinicopathological risk factors of oral second primary tumours. Oral Oncol. Rep..

[20] Bonetti Valente V., Mantovan Mazzon B., Urbano Collado F., Conrado Neto S., Lucia Marcal Mazza Sundefeld M., Ricardo Biasoli E., Issamu Miyahara G., Galera Bernabe D. (2022). Clinicopathological and prognostic profile of non-smoking and non-drinking head and neck cancer patients: A population-based comparative study. Oral Oncol..

[21] Koo K., Barrowman R., McCullough M., Iseli T., Wiesenfeld D. (2013). Non-smoking non-drinking elderly females: A clinically distinct subgroup of oral squamous cell carcinoma patients. Int. J. Oral Maxillofac. Surg..

[22] Uddin S., Singh A., Mishra V., Agrawal N., Gooi Z., Izumchenko E. (2022). Molecular drivers of oral cavity squamous cell carcinoma in non-smoking and non-drinking patients: What do we know so far?. Oncol. Rev..

[23] Laniosz V., Torgerson R.R., Ramos-Rodriguez A.J., Ma J.E., Mara K.C., Weaver A.L., Bruce A.J. (2019). Incidence of squamous cell carcinoma in oral lichen planus: A 25-year population-based study. Int. J. Dermatol..

[24] Querzoli G., Gabusi A., Gissi D.B., Bassani S., Rossi R., Tarsitano A., Montebugnoli L., Foschini M.P. (2025). Cell-mediated mucositis of the oral cavity: Narrative review on etiology, clinico-pathological aspects and malignant transformation. Pathologica.

[25] Bouaoud J., Foy J.P., Tortereau A., Michon L., Lavergne V., Gadot N., Boyault S., Valantin J., De Souza G., Zrounba P. (2021). Early changes in the immune microenvironment of oral potentially malignant disorders reveal an unexpected association of M2 macrophages with oral cancer free survival. Oncoimmunology.

[26] Tenore G., Mohsen A., Rocchetti F., Rossi G., Cassoni A., Battisti A., Della Monaca M., Di Gioia C.R.T., De Felice F., Botticelli A. (2023). Risk of Oral Squamous Cell Carcinoma in One Hundred Patients with Oral Lichen Planus: A Follow-Up Study of Umberto I University Hospital of Rome. Cancers.

[27] Ali A., Molska G.R., Yeo H., Esfandiari N., Jeong W., Huang M., Magalhaes M. (2025). Immune Microenvironment in Oral Potentially Malignant Disorders and Oral Cancer: A Narrative Review. Int. J. Mol. Sci..

[28] Salminen A. (2022). Clinical perspectives on the age-related increase of immunosuppressive activity. J. Mol. Med..

