# Supplementary material for: Distinct Second Primary Tumor Phenotypes in Oral Squamous Cell Carcinoma According to Exposure Status and Immune Background
Source: J Clin Med. 2026 Feb 16;15(4):1563. doi: 10.3390/jcm15041563 (PMC12941459; doi:10.3390/jcm15041563)
Supplement: Supplementary file 1 [file jcm-15-01563-s001.zip › jcm-4118283-supplementary.pdf]

Supplementary Tables

Table S1. Median follow-up duration across key strata.

Comparison	Category	Follow-up, months, median (IQR)	p-value
Exposure status	NSND (n = 84)	74.5 (16.0–107.0)	
	SD (n = 158)	49.0 (17.0–100.5)	0.258
Immune-modulating disease	Present (n = 85)	75.0 (20.0–98.0)	
	Absent (n = 157)	50.0 (15.0–108.0)	0.483
Oral lichen planus	Present (n = 20)	94.0 (77.8–111.2)	
	Absent (n = 222)	48.0 (15.0–100.8)	0.001

NSND, non-smoker/non-drinker; SD, smoker and/or drinker; **p-values** were calculated using the Mann–Whitney U test.

Table S2. Overlap between eoSPT and mOSCC phenotypes.

Category	n	%
No SPT	160	66.1%
eoSPT only	54	22.3%
mOSCC only	25	10.3%
Both mOSCC + eoSPT	3	1.2%

Note: This table presents a non–mutually exclusive accounting of SPT phenotypes. In the primary analyses, eoSPT and mOSCC were evaluated using a prespecified mutually exclusive hierarchical classification.

Table S3. Sensitivity logistic regression models for eoSPT.

Model	Predictor	OR (95% CI)	p-value
**Original model**	NSND (vs SD)	0.37 (0.15–0.96)	0.041
	Age (per 10 years)	1.34 (1.03–1.74)	0.031
	Female sex	0.23 (0.08–0.66)	0.006
	Any immune-modulating condition (yes vs no)	1.83 (0.82–4.06)	0.141
**Original + follow-up**	NSND (vs SD)	0.39 (0.15–1.01)	0.052
	Age (per 10 years)	1.31 (1.00–1.71)	0.048
	Female sex	0.24 (0.08–0.68)	0.007
	Any immune-modulating condition (yes vs no)	1.82 (0.82–4.07)	0.143
	Follow-up duration (per year)	0.96 (0.89–1.04)	0.377
**Original + follow-up + stage**	NSND (vs SD)	0.35 (0.13–0.94)	0.038
	Age (per 10 years)	1.33 (1.01–1.74)	0.042
	Female sex	0.24 (0.08–0.69)	0.008
	Any immune-modulating condition (yes vs no)	1.95 (0.85–4.45)	0.114
	Follow-up duration (per year)	0.97 (0.89–1.06)	0.511
	Stage II (vs I)	0.69 (0.19–2.49)	0.567
	Stage III (vs I)	0.62 (0.17–2.26)	0.466
	Stage IVa (vs I)	0.62 (0.19–2.05)	0.430
	Stage IVb (vs I)	1.22 (0.35–4.25)	0.752
**eoSPT_any (incl. overlap)**	NSND (vs SD)	0.46 (0.19–1.11)	0.083
	Age (per 10 years)	1.25 (0.97–1.60)	0.084
	Female sex	0.25 (0.09–0.67)	0.006
	Any immune-modulating condition (yes vs no)	1.89 (0.87–4.10)	0.108

eoSPT, extra-oral second primary tumour; NSND, never-smoker/never-drinker; SD, smoker and/or drinker. Note: Sensitivity analyses assessing the impact of follow-up adjustment, tumour stage, and non–mutually exclusive phenotype definition.

Table S4. Sensitivity logistic regression models for mOSCC.

Model	Predictor	OR (95% CI)	p-value
**Original model**	NSND (vs SD)	1.30 (0.46–3.67)	0.625
	Age (per 10 years)	1.37 (1.01–1.84)	0.042
	Female sex	1.11 (0.38–3.20)	0.851
	Oral lichen planus (yes vs no)	3.47 (1.04–11.52)	0.043
**Original + follow-up**	NSND (vs SD)	1.21 (0.43–3.42)	0.721
	Age (per 10 years)	1.48 (1.07–2.04)	0.017
	Female sex	1.10 (0.38–3.18)	0.864
	Oral lichen planus (yes vs no)	2.71 (0.78–9.43)	0.117
	Follow-up duration (per year)	1.09 (0.98–1.21)	0.115
**Original + follow-up + stage**	NSND (vs SD)	1.30 (0.46–3.72)	0.622
	Age (per 10 years)	1.49 (1.08–2.07)	0.016
	Female sex	1.11 (0.37–3.28)	0.851
	Oral lichen planus (yes vs no)	2.87 (0.77–10.79)	0.118
	Follow-up duration (per year)	1.07 (0.96–1.20)	0.215
	Stage II (vs I)	2.18 (0.52–9.09)	0.284
	Stage III (vs I)	2.15 (0.47–9.87)	0.326
	Stage IVa (vs I)	0.85 (0.19–3.74)	0.827
	Stage IVb (vs I)	0.94 (0.18–4.92)	0.937
**Firth penalized**	NSND (vs SD)	1.33 (0.48–3.66)	0.579
	Age (per 10 years)	1.35 (1.00–1.80)	0.047
	Female sex	1.13 (0.40–3.18)	0.812
	Oral lichen planus (yes vs no)	3.31 (1.01–10.78)	0.047

mOSCC, multifocal oral squamous cell carcinoma; NSND, non-smoker/non-drinker; SD, smoker and/or drinker; OLP, oral lichen planus.
Note: OR (95% CI) from logistic regression. Age scaled per 10 years. Follow-up duration scaled per year. Stage was included as a categorical covariate (reference = Stage I) in stage-adjusted models. The Firth penalized model uses bias-reduced estimation.

Table S5. Exploratory mOSCC model restricted to NSND patients.

Model	Predictor	OR (95% CI)	p-value
**NSND-only**	Age (per 10 years)	1.26 (0.86–1.85)	0.232
	Female sex	0.43 (0.10–1.78)	0.244
	OLP (yes vs no)	5.87 (1.48–23.34)	0.012

mOSCC, multifocal oral squamous cell carcinoma; NSND, non-smoker/non-drinker; OLP, oral lichen planus.
Note: Model fitted only among NSND patients to evaluate whether the association between OLP and mOSCC persists in the absence of tobacco and alcohol exposure. Age scaled per 10 years.
